# 2-(3-Nitro­phen­oxy)quinoxaline

**DOI:** 10.1107/S1600536810034100

**Published:** 2010-08-28

**Authors:** Noor Doha Hassan, Zanariah Abdullah, Hairul Anuar Tajuddin, Zainal A. Fairuz, Seik Weng Ng, Edward R. T. Tiekink

**Affiliations:** a Department of Chemistry, University of Malaya, 50603 Kuala Lumpur, Malaysia

## Abstract

In the title mol­ecule, C_14_H_9_N_3_O_3_, the dihedral angle between the quinoxaline and benzene rings is 77.13 (9)°. The mol­ecule is twisted about the ether–benzene O—C bond, with a C—O—C—C torsion angle of −102.8 (2)°. In the crystal, mol­ecules are linked by C—H⋯O hydrogen bonds, forming layers in the *ab* plane, with one nitro O atom accepting two such inter­actions. The layers stack along the *c*-axis direction *via* weak C—H⋯π inter­actions.

## Related literature

For background to the fluorescence properties of compounds related to the title compound, see: Kawai *et al.* (2001 ▶); Abdullah (2005 ▶). For the structures of the polymorphic phenyl quinoxalin-2-yl ether compound, see: Hassan *et al.* (2008 ▶); Abdullah & Ng (2008 ▶).
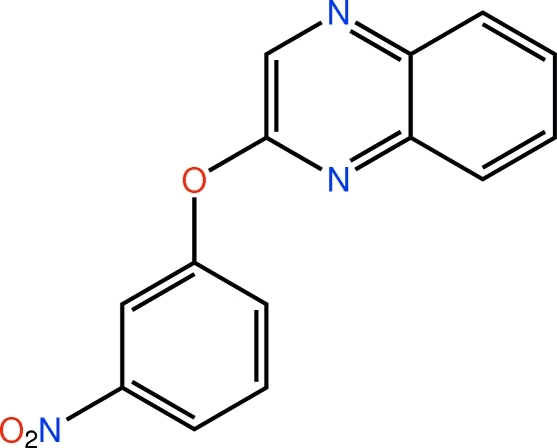


## Experimental

### 

#### Crystal data

C_14_H_9_N_3_O_3_
*M* *_r_* = 267.24
Monoclinic, 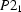

*a* = 6.0643 (6) Å
*b* = 5.3676 (5) Å
*c* = 18.2443 (17) Å
β = 91.780 (1)°
*V* = 593.58 (10) Å^3^
*Z* = 2
Mo *K*α radiation
μ = 0.11 mm^−1^
*T* = 100 K
0.35 × 0.25 × 0.15 mm

#### Data collection

Bruker SMART APEX CCD diffractometer
5637 measured reflections
1502 independent reflections
1403 reflections with *I* > 2σ(*I*)
*R*_int_ = 0.028

#### Refinement

*R*[*F* ^2^ > 2σ(*F* ^2^)] = 0.034
*wR*(*F* ^2^) = 0.093
*S* = 1.04
1501 reflections
181 parameters
1 restraint
H-atom parameters constrained
Δρ_max_ = 0.24 e Å^−3^
Δρ_min_ = −0.22 e Å^−3^

### 

Data collection: *APEX2* (Bruker, 2009 ▶); cell refinement: *SAINT* (Bruker, 2009 ▶); data reduction: *SAINT*; program(s) used to solve structure: *SHELXS97* (Sheldrick, 2008 ▶); program(s) used to refine structure: *SHELXL97* (Sheldrick, 2008 ▶); molecular graphics: *ORTEP-3* (Farrugia, 1997 ▶) and *DIAMOND* (Brandenburg, 2006 ▶); software used to prepare material for publication: *publCIF* (Westrip, 2010 ▶).

## Supplementary Material

Crystal structure: contains datablocks global, I. DOI: 10.1107/S1600536810034100/hb5615sup1.cif

Structure factors: contains datablocks I. DOI: 10.1107/S1600536810034100/hb5615Isup2.hkl

Additional supplementary materials:  crystallographic information; 3D view; checkCIF report

## Figures and Tables

**Table 1:** Hydrogen-bond geometry (Å, °) *Cg*1 is the centroid of the C3–C8 ring.

*D*—H⋯*A*	*D*—H	H⋯*A*	*D*⋯*A*	*D*—H⋯*A*
C10—H10⋯O2^i^	0.95	2.34	3.282 (3)	173
C12—H12⋯O2^ii^	0.95	2.44	3.159 (2)	133
C5—H5⋯*Cg*1^iii^	0.95	2.99	3.696 (2)	133

Symmetry codes: (i) 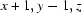; (ii) 
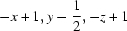
; (iii) 
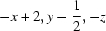
.

**Fig. 1.**
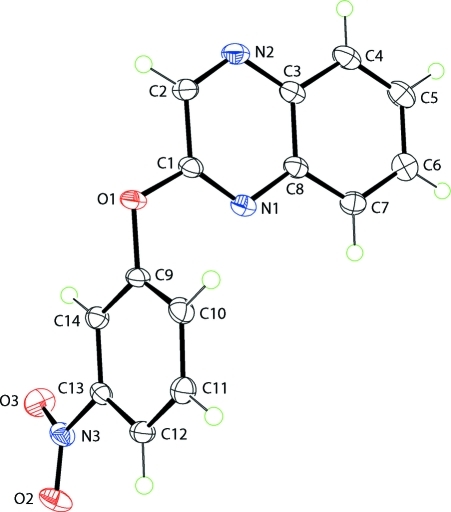
The molecular structure of (I) showing displacement ellipsoids at the 50% probability level.

**Fig. 2.**
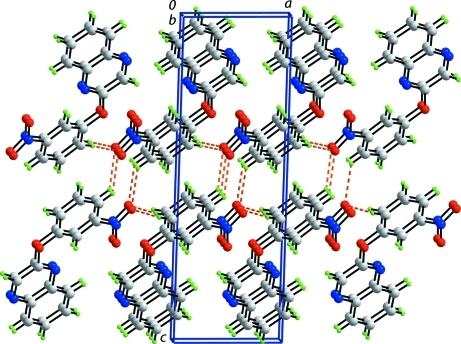
Supramolecular layer in the ab plane mediated by C–H···O interactions, shown as orange dashed lines, in (I).

**Fig. 3.**
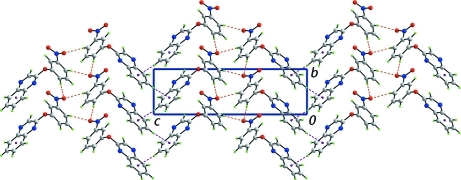
Unit-cell contents shown in projection down the a axis in (I), highlighting the stacking of layers along the c direction. The C–H···O and C–H···π interactions are shown as orange and purple dashed lines, respectively.

## supplementary crystallographic information

### Comment

Quinoxaline derivatives show interesting fluorescence properties (Kawai *et al.* 2001; Abdullah, 2005) and this observation prompted the synthesis and characterization of the title compound, (I).

The molecule in (I), Fig. 1, is bent as the quinoxaline ring [r.m.s. deviation = 0.025 Å] forms a dihedral angle of 77.13 (9) ° with the benzene molecule. The twist in the molecule is seen in the value of the C1–O1–C9–C14 torsion angle of -102.8 (2) °. Overall the conformation of the molecule matches those found in the polymorphic phenyl quinoxalin-2-yl ether compound (Hassan *et al.*, 2008; Abdullah & Ng, 2008). In (I), the nitro group is slightly twisted out of the plane of the benzene ring to which it is bonded as seen in the O2–N3–C13–C12 torsion angle of 12.6 (3) °.

The bifurcated nitro-O2 atom is pivotal in the crystal packing, forming two close C–H···O interactions, Table 1, leading to the formation of layers in the *ab* plane, Fig. 2. These stack along the *c* axis, being connected by C–H···π interactions, Fig. 3.

### Experimental

3-Nitrophenol (5 mmol) was dissolved in tetrahydrofuran (100 ml) to which was added 2-chloroquinoxaline with a stoichiometric amount of NaOH. The solution was refluxed for 4 h. The mixture was extracted using 5% sodium hydroxide solution (5 ml), then chloroform (20 ml), washed with distilled water (30 ml), and dried over anhydrous sodium hydroxide. Evaporation of the solvent gave a red solid and recrystallization was from its ethanol solution to yield red prisms of (I).

### Refinement

Carbon-bound H-atoms were placed in calculated positions (C—H 0.95 Å) and were included in the refinement in the riding model approximation, with *U*_iso_(H) set to 1.2*U*_equiv_(C). In the absence of significant anomalous scattering effects, 1199 Friedel pairs were averaged in the final refinement. In the final refinement a low angle reflection evidently effected by the beam stop were omitted, *i.e*. 0 0 1.

### Crystal data

**Table:**

C_14_H_9_N_3_O_3_	*F*(000) = 276
*M**_r_* = 267.24	*D*_x_ = 1.495 Mg m^−^^3^
Monoclinic, *P*2_1_	Mo *K*α radiation, λ = 0.71073 Å
Hall symbol: P 2yb	Cell parameters from 2560 reflections
*a* = 6.0643 (6) Å	θ = 2.2–28.3°
*b* = 5.3676 (5) Å	µ = 0.11 mm^−^^1^
*c* = 18.2443 (17) Å	*T* = 100 K
β = 91.780 (1)°	Prism, red
*V* = 593.58 (10) Å^3^	0.35 × 0.25 × 0.15 mm
*Z* = 2	

### Data collection

**Table:**

Bruker SMART APEX CCD diffractometer	1403 reflections with *I* > 2σ(*I*)
Radiation source: fine-focus sealed tube	*R*_int_ = 0.028
graphite	θ_max_ = 27.5°, θ_min_ = 1.1°
ω scans	*h* = −7→7
5637 measured reflections	*k* = −6→6
1502 independent reflections	*l* = −23→23

### Refinement

**Table:**

Refinement on *F*^2^	Secondary atom site location: difference Fourier map
Least-squares matrix: full	Hydrogen site location: inferred from neighbouring sites
*R*[*F*^2^ > 2σ(*F*^2^)] = 0.034	H-atom parameters constrained
*wR*(*F*^2^) = 0.093	*w* = 1/[σ^2^(*F*_o_^2^) + (0.0584*P*)^2^ + 0.098*P*] where *P* = (*F*_o_^2^ + 2*F*_c_^2^)/3
*S* = 1.04	(Δ/σ)_max_ < 0.001
1501 reflections	Δρ_max_ = 0.24 e Å^−^^3^
181 parameters	Δρ_min_ = −0.22 e Å^−^^3^
1 restraint	Absolute structure: nd
Primary atom site location: structure-invariant direct methods	

### Special details

**Table:**

Geometry. All s.u.'s (except the s.u. in the dihedral angle between two l.s. planes) are estimated using the full covariance matrix. The cell s.u.'s are taken into account individually in the estimation of s.u.'s in distances, angles and torsion angles; correlations between s.u.'s in cell parameters are only used when they are defined by crystal symmetry. An approximate (isotropic) treatment of cell s.u.'s is used for estimating s.u.'s involving l.s. planes.
Refinement. Refinement of *F*^2^ against ALL reflections. The weighted *R*-factor *wR* and goodness of fit *S* are based on *F*^2^, conventional *R*-factors *R* are based on *F*, with *F* set to zero for negative *F*^2^. The threshold expression of *F*^2^ > 2σ(*F*^2^) is used only for calculating *R*-factors(gt) *etc*. and is not relevant to the choice of reflections for refinement. *R*-factors based on *F*^2^ are statistically about twice as large as those based on *F*, and *R*- factors based on ALL data will be even larger.

### Fractional atomic coordinates and isotropic or equivalent isotropic displacement parameters (Å^2^)

**Table:**

	*x*	*y*	*z*	*U*_iso_*/*U*_eq_	
O1	1.2692 (2)	0.5001 (3)	0.29144 (7)	0.0241 (3)	
O2	0.4391 (2)	0.8540 (4)	0.41409 (8)	0.0309 (4)	
O3	0.5748 (2)	0.9758 (3)	0.31197 (9)	0.0309 (4)	
N1	1.1038 (2)	0.1916 (4)	0.22012 (8)	0.0203 (4)	
N2	1.4817 (3)	0.2107 (4)	0.13226 (9)	0.0242 (4)	
N3	0.5762 (3)	0.8419 (4)	0.36598 (9)	0.0224 (4)	
C1	1.2685 (3)	0.3411 (4)	0.23292 (10)	0.0201 (4)	
C2	1.4591 (3)	0.3556 (4)	0.18864 (11)	0.0229 (4)	
H2	1.5713	0.4735	0.2007	0.028*	
C3	1.3147 (3)	0.0427 (4)	0.11749 (10)	0.0212 (4)	
C4	1.3319 (3)	−0.1261 (5)	0.05877 (11)	0.0261 (5)	
H4	1.4576	−0.1216	0.0290	0.031*	
C5	1.1678 (3)	−0.2970 (5)	0.04435 (11)	0.0281 (5)	
H5	1.1817	−0.4113	0.0050	0.034*	
C6	0.9785 (3)	−0.3042 (5)	0.08741 (11)	0.0268 (5)	
H6	0.8666	−0.4243	0.0774	0.032*	
C7	0.9563 (3)	−0.1377 (5)	0.14384 (10)	0.0227 (4)	
H7	0.8263	−0.1393	0.1716	0.027*	
C8	1.1248 (3)	0.0355 (4)	0.16091 (10)	0.0194 (4)	
C9	1.0867 (3)	0.4856 (4)	0.33663 (10)	0.0198 (4)	
C10	1.0743 (3)	0.2998 (4)	0.38865 (11)	0.0228 (4)	
H10	1.1866	0.1769	0.3930	0.027*	
C11	0.8952 (3)	0.2945 (4)	0.43471 (11)	0.0229 (4)	
H11	0.8867	0.1690	0.4712	0.027*	
C12	0.7295 (3)	0.4715 (4)	0.42751 (10)	0.0200 (4)	
H12	0.6051	0.4671	0.4579	0.024*	
C13	0.7504 (3)	0.6547 (4)	0.37477 (10)	0.0181 (4)	
C14	0.9282 (3)	0.6693 (4)	0.32869 (10)	0.0193 (4)	
H14	0.9402	0.7989	0.2936	0.023*	

### Atomic displacement parameters (Å^2^)

**Table:**

	*U*^11^	*U*^22^	*U*^33^	*U*^12^	*U*^13^	*U*^23^
O1	0.0166 (6)	0.0277 (8)	0.0286 (7)	−0.0011 (6)	0.0085 (5)	−0.0071 (7)
O2	0.0254 (7)	0.0371 (9)	0.0307 (8)	0.0124 (7)	0.0081 (6)	−0.0029 (7)
O3	0.0265 (7)	0.0290 (9)	0.0372 (8)	0.0069 (7)	0.0003 (6)	0.0113 (7)
N1	0.0184 (7)	0.0230 (9)	0.0198 (7)	0.0034 (7)	0.0028 (6)	0.0005 (7)
N2	0.0211 (8)	0.0274 (10)	0.0244 (8)	0.0027 (7)	0.0074 (6)	0.0035 (8)
N3	0.0192 (7)	0.0220 (9)	0.0260 (8)	0.0050 (7)	0.0011 (6)	−0.0017 (8)
C1	0.0166 (8)	0.0204 (10)	0.0233 (9)	0.0038 (8)	0.0033 (7)	0.0004 (9)
C2	0.0188 (9)	0.0241 (11)	0.0262 (10)	0.0003 (9)	0.0056 (7)	0.0011 (9)
C3	0.0222 (9)	0.0231 (11)	0.0183 (9)	0.0061 (8)	0.0035 (7)	0.0025 (8)
C4	0.0285 (10)	0.0294 (12)	0.0208 (9)	0.0081 (10)	0.0062 (7)	0.0013 (9)
C5	0.0327 (11)	0.0308 (12)	0.0210 (9)	0.0080 (10)	0.0006 (8)	−0.0042 (9)
C6	0.0286 (10)	0.0272 (12)	0.0245 (10)	0.0019 (9)	−0.0024 (8)	−0.0005 (10)
C7	0.0227 (9)	0.0262 (11)	0.0193 (9)	0.0010 (9)	0.0017 (7)	0.0013 (9)
C8	0.0196 (8)	0.0211 (11)	0.0177 (8)	0.0045 (8)	0.0018 (6)	0.0026 (8)
C9	0.0134 (8)	0.0234 (10)	0.0228 (9)	−0.0010 (8)	0.0041 (6)	−0.0058 (9)
C10	0.0182 (9)	0.0205 (11)	0.0296 (10)	0.0048 (8)	−0.0001 (7)	−0.0018 (8)
C11	0.0244 (9)	0.0202 (11)	0.0240 (10)	0.0004 (8)	0.0016 (7)	0.0023 (8)
C12	0.0176 (8)	0.0228 (11)	0.0198 (9)	0.0003 (8)	0.0032 (6)	−0.0015 (8)
C13	0.0167 (8)	0.0181 (10)	0.0196 (8)	0.0025 (7)	0.0003 (6)	−0.0032 (8)
C14	0.0193 (9)	0.0202 (10)	0.0186 (8)	−0.0001 (8)	0.0014 (6)	−0.0007 (8)

### Geometric parameters (Å, °)

**Table:**

O1—C1	1.367 (2)	C5—H5	0.9500
O1—C9	1.402 (2)	C6—C7	1.373 (3)
O2—N3	1.229 (2)	C6—H6	0.9500
O3—N3	1.219 (2)	C7—C8	1.410 (3)
N1—C1	1.297 (3)	C7—H7	0.9500
N1—C8	1.376 (3)	C9—C10	1.380 (3)
N2—C2	1.300 (3)	C9—C14	1.382 (3)
N2—C3	1.376 (3)	C10—C11	1.394 (3)
N3—C13	1.463 (2)	C10—H10	0.9500
C1—C2	1.433 (2)	C11—C12	1.386 (3)
C2—H2	0.9500	C11—H11	0.9500
C3—C4	1.409 (3)	C12—C13	1.384 (3)
C3—C8	1.419 (2)	C12—H12	0.9500
C4—C5	1.373 (3)	C13—C14	1.390 (3)
C4—H4	0.9500	C14—H14	0.9500
C5—C6	1.411 (3)		
			
C1—O1—C9	116.22 (15)	C6—C7—C8	120.51 (18)
C1—N1—C8	115.37 (16)	C6—C7—H7	119.7
C2—N2—C3	116.86 (16)	C8—C7—H7	119.7
O3—N3—O2	123.90 (18)	N1—C8—C7	119.41 (16)
O3—N3—C13	118.70 (16)	N1—C8—C3	121.23 (17)
O2—N3—C13	117.40 (17)	C7—C8—C3	119.35 (18)
N1—C1—O1	120.68 (16)	C10—C9—C14	122.35 (16)
N1—C1—C2	124.22 (18)	C10—C9—O1	120.34 (17)
O1—C1—C2	115.10 (17)	C14—C9—O1	117.25 (18)
N2—C2—C1	121.36 (19)	C9—C10—C11	119.30 (18)
N2—C2—H2	119.3	C9—C10—H10	120.3
C1—C2—H2	119.3	C11—C10—H10	120.3
N2—C3—C4	119.93 (17)	C12—C11—C10	120.34 (19)
N2—C3—C8	120.91 (17)	C12—C11—H11	119.8
C4—C3—C8	119.17 (18)	C10—C11—H11	119.8
C5—C4—C3	120.34 (18)	C13—C12—C11	118.07 (17)
C5—C4—H4	119.8	C13—C12—H12	121.0
C3—C4—H4	119.8	C11—C12—H12	121.0
C4—C5—C6	120.6 (2)	C12—C13—C14	123.39 (18)
C4—C5—H5	119.7	C12—C13—N3	118.84 (16)
C6—C5—H5	119.7	C14—C13—N3	117.76 (18)
C7—C6—C5	120.0 (2)	C9—C14—C13	116.51 (18)
C7—C6—H6	120.0	C9—C14—H14	121.7
C5—C6—H6	120.0	C13—C14—H14	121.7
			
C8—N1—C1—O1	−178.20 (17)	C4—C3—C8—N1	178.05 (18)
C8—N1—C1—C2	2.1 (3)	N2—C3—C8—C7	179.52 (18)
C9—O1—C1—N1	1.7 (3)	C4—C3—C8—C7	−0.8 (3)
C9—O1—C1—C2	−178.60 (17)	C1—O1—C9—C10	79.9 (2)
C3—N2—C2—C1	−0.3 (3)	C1—O1—C9—C14	−102.8 (2)
N1—C1—C2—N2	−1.8 (3)	C14—C9—C10—C11	0.5 (3)
O1—C1—C2—N2	178.42 (18)	O1—C9—C10—C11	177.72 (17)
C2—N2—C3—C4	−177.78 (19)	C9—C10—C11—C12	1.1 (3)
C2—N2—C3—C8	1.9 (3)	C10—C11—C12—C13	−1.5 (3)
N2—C3—C4—C5	178.96 (19)	C11—C12—C13—C14	0.3 (3)
C8—C3—C4—C5	−0.7 (3)	C11—C12—C13—N3	179.51 (17)
C3—C4—C5—C6	0.8 (3)	O3—N3—C13—C12	−167.50 (18)
C4—C5—C6—C7	0.7 (3)	O2—N3—C13—C12	12.5 (3)
C5—C6—C7—C8	−2.2 (3)	O3—N3—C13—C14	11.8 (3)
C1—N1—C8—C7	178.47 (18)	O2—N3—C13—C14	−168.20 (18)
C1—N1—C8—C3	−0.4 (3)	C10—C9—C14—C13	−1.7 (3)
C6—C7—C8—N1	−176.59 (19)	O1—C9—C14—C13	−178.92 (16)
C6—C7—C8—C3	2.3 (3)	C12—C13—C14—C9	1.3 (3)
N2—C3—C8—N1	−1.6 (3)	N3—C13—C14—C9	−177.99 (16)

### Hydrogen-bond geometry (Å, °)

**Table:**

Cg1 is the centroid of the C3–C8 ring.

**Table:**

*D*—H···*A*	*D*—H	H···*A*	*D*···*A*	*D*—H···*A*
C10—H10···O2^i^	0.95	2.34	3.282 (3)	173
C12—H12···O2^ii^	0.95	2.44	3.159 (2)	133
C5—H5···Cg1^iii^	0.95	2.99	3.696 (2)	133

Symmetry codes: (i) *x*+1, *y*−1, *z*; (ii) −*x*+1, *y*−1/2, −*z*+1; (iii) −*x*+2, *y*−1/2, −*z*.
